# Sociodemographic determinants and health outcome variation in individuals with type 1 diabetes mellitus: A register-based study

**DOI:** 10.1371/journal.pone.0199170

**Published:** 2018-06-29

**Authors:** Carl Willers, Hanna Iderberg, Mette Axelsen, Tobias Dahlström, Bettina Julin, Janeth Leksell, Agneta Lindberg, Peter Lindgren, Karin Looström Muth, Ann-Marie Svensson, Mikael Lilja

**Affiliations:** 1 Dept for Clinical Science and Education, Karolinska Institutet, Stockholm, Sweden; 2 Ivbar Institute, Stockholm, Sweden; 3 Medical Management Centre, Karolinska Institutet, Stockholm, Sweden; 4 Department of Internal Medicine at Institute of Medicine, University of Gothenburg, Gothenburg, Sweden; 5 Department of Public Health and Caring Sciences, Health Services Research, Uppsala university, Uppsala, Sweden; 6 School of Education, Health and Social Studies, Dalarna University, Dalarna and Clinical diabetology and metabolism, Department of medical sciences, Uppsala University, Uppsala, Sweden; 7 Hässleholm Hospital, Hässleholm, Sweden; 8 The Swedish Institute for Health Economics, Lund, Sweden; 9 Department of Internal Medicine, Alingsås Hospital, Alingsås, Sweden; 10 The Swedish National Diabetes Register, Västra Götalandsregionen, Gothenburg, Sweden; 11 Department of Public Health and Clinical Medicine, Unit of Research, Education, and Development, Östersund Hospital, Umeå University, Umeå, Sweden; University of Tennessee Health Science Center, UNITED STATES

## Abstract

**Background:**

Socioeconomic status, origin or demographic attributes shall not determine the quality of healthcare delivery, according to e.g. United Nations and European Union rules. Health equity has been defined as the absence of systematic disparities and unwarranted differences between groups defined by differences in social advantages. A study was performed to investigate whether this was applicable to type 1 diabetes mellitus (T1D) care in a setting with universal, tax-funded healthcare.

**Methods:**

This retrospective registry-study was based on patient-level data from individuals diagnosed with T1D during 2010–2011 (n = 16,367) in any of seven Swedish county councils (covering ~65% of the Swedish population). Health equity in T1D care was analysed through multivariate regression analyses on absolute HbA1c level at one-year follow-up, one-year change in estimated glomerular filtration rate (eGFR) and one-year change in cardiovascular risk score, using selected sociodemographic dimensions as case-mix factors.

**Results:**

Higher educational level was consistently associated with lower levels of HbA1c, and so was being married. Never married was associated with worse eGFR development, and lower educational level was associated with higher cardiovascular risk. Women had higher HbA1c levels than men, and glucose control was significantly worse in patients below the age of 25.

**Conclusion:**

Patients’ sociodemographic profile was strongly associated with absolute levels of risk factor control in T1D, but also with an increased annual deterioration in eGFR. Whether these systematic differences stem from patient-related problems or healthcare organisational shortcomings is a matter for further research. The results, though, highlight the need for intensified diabetes management education and secondary prevention directed towards T1D patients, taking sociodemographic characteristics into account.

## Background

Healthcare delivery shall be equitable and independent of socioeconomic status, origin or demographic attributes, as stated in e.g. UN and EU rules as well as in Swedish law.[1–3] Health equity has been defined as the absence of systematic disparities between groups defined by differences in social advantages.[4] Thus, health care delivery should be executed in accordance with individuals’ need for good health without any unwarranted differences in health outcomes dependent on differences in sociodemographic patient characteristics. Any differences in health outcomes dependent on differences in sociodemographic characteristics are important to understand comprehensively to determine how different individuals with type 1 diabetes mellitus (T1D) should be treated for optimal results.

Scientific literature point to differences in diabetes care between sociodemographic groups in various settings in terms of differences in HbA1c and cardiovascular disease (CVD) risk,[5–7] even though the lion’s share of literature on the subject treats type 2 diabetes mellitus (T2D). Individuals with T1D are generally younger at diagnosis compared to T2D, and consequently the time horizon for impacting health outcomes in T1D individuals is longer. Depending on treatment efficiency, the impact per individual over time could therefore be expected to be larger in T1D.

For the patient population studied here, the conditions for equal health outcomes–one general principal of health equity[8]–are good; Sweden has a system of universal, tax-funded, access to acute health care and inpatient care. A major share of the primary care visit fee is also tax-funded.[9] Nevertheless, there are differences between groups, as have been presented previously.[10] Furthermore, the conditions are good for studying how health outcomes may differ dependent on sociodemographic characteristics, as national registers track socioeconomic and demographic data as well as disease-specific outcome measures. Findings pointing to that certain sociodemographic characteristics play a significant role in T1D treatment could be of help in modulating treatments and/or instructions to fit these patient characteristics better. With the present study, we do not intend to determine whether any potential differences observed are unwarranted or not but to present facts and take part in emphasizing the importance of keeping this topic on the agenda, even–or perhaps in particular–in countries with universal, tax-funded access to healthcare.

Sociodemographic drivers to differences in survival, risk factor control and CVD in T1D patients (also Swedish) have been presented.[10] We set out to expand on these findings by also studying one-year changes in kidney function–estimated Glomerular Filtration Rate (eGFR)–and development of CVD risk in T1D patients and its association with sociodemographic characteristics, comorbidities and other clinically relevant factors. Furthermore, this study set out to control for additional relevant social risk factors (e.g. disability pension and sick-leave history).

The objective of this study was to investigate which sociodemographic factors potentially drive differences in health outcomes among Swedish T1D patients, regarding levels of HbA1c, and one-year change in eGFR and CVD risk, respectively.

## Material and methods

### Study population and data sources

Sveus is a research collaboration in which seven Swedish regions develop systems for value-based monitoring and reimbursement of health care, initially funded by the Ministry of Health and Social affairs. The research within Sveus diabetes is based on the extended research group (Sveus, www.sveus.se) representing county councils, patient organization, specialists, diabetes nurses, quality registry and Ivbar Institute (R&D company).

A retrospective registry study was conducted based on data from individuals of 18 years of age or older with T1D registered during 2010–2011 in the administrative systems of seven Swedish county councils (Dalarna, Jämtland Härjedalen, Skåne, Stockholm, Uppsala, Västra Götaland and Östergötland), covering ~65% of the Swedish population. T1D was defined by diagnosis registration within the regions’ patient administrative systems together with diagnosis registration in the National Diabetes Register (NDR, with a coverage rate of almost 90% at the time and data provided by nurses and physicians trained in registering procedures)[11] and/or use of insulin according to the Prescribed Drug Registry (PDR, a national registry tracking all filled prescriptions).

To include only previously knowns cases of T1D, patients diagnosed during the last year were excluded. Data from the administrative systems were linked on patient level (based on unique, anonymized social security numbers) to data from the NDR, socioeconomic data from Statistics Sweden, data on filled prescriptions from the PDR and data on sick-leave and disability pension from the Swedish Social Insurance Agency.

The study was approved by the Regional Ethical Review Board in Stockholm (2013/1197-31) and conducted in accordance with the Declaration of Helsinki. Oral or written informed consent has been obtained from each patient participating in NDR. A flowchart depicting the study design is presented in supplementary S1 Fig.

### Outcomes

Three indicators were used to study health outcomes; absolute HbA1c level, one-year change in eGFR and one-year change in CVD risk score. These indicators were chosen to enable comprehensive understanding of risk factors in terms of absolute (HbA1c) levels as well as development over time, and their association to sociodemographic characteristics. Analyzed health outcomes were subject to data availability within existing registries and selected based on literature and expertise of the research group. Data on all three indicators came from the NDR.

Higher levels of glycated hemoglobin (HbA1c) indicate higher risk for complications and worse prognosis and is often used as indicator for quality of diabetes care.[12,13].

eGFR is a measure of kidney function, but also a marker of cardiovascular risk and mortality.[14,15] For additional understanding on how the kidney function develop over the course of disease, one-year change in eGFR was studied. eGFR was estimated on patient level through the revised Malmö-Lund formula[16]. One-year change was computed through subtraction of eGFR at the end of the year with eGFR at baseline.

To predict the 5-year risk for cardiovascular events in T1D patients, Cederholm et al. has developed an algorithm based on NDR real-life data,[17] including eight different variables: age at diagnosis, duration of diabetes, total-cholesterol/HDL–cholesterol ratio, HbA1c, systolic blood pressure, smoking status, macro albuminuria and previous heart conditions (last two years). This algorithm was used to compute the risk score on patient level. Only patients with input values within the following intervals were included, in accordance with Cederholm et al.[17]: Body Mass Index (BMI) of 16–50, creatinine of 20–800 μmol/l, and remaining input values had to be registered in the interval of ±3 months from baseline to be considered valid. Previous heart conditions were defined by ICD-10 codes as stated in supplementary S1 Table.

### Case-mix factors

To analyze the association between sociodemographic factors and health outcomes in T1D patients, it was of importance to adjust for other relevant factors potentially differing systematically between sociodemographic groups. Case-mix factors were identified, selected and considered relevant based on literature and clinical expertise of the multi-professional expert group, and are presented in Table 1.

**Table 1:** Descriptive statistics of study sample at baseline. Standard deviation in parentheses.

Variable	Average (std.dev.)
Age (years)	50.1 (16.1)
Female (%)	37.9
Education (highest level)	
- Comprehensive (≤9 years) (%)	20.3
- High school (9–12 years) (%)	49.4
- College/university (≥12 years) (%)	30.3
Civil status	
- Married (%)	46.7
- Never married (%)	36.7
- Divorced (%)	12.9
- Widowed (%)	3.7
Region of birth	
- Nordic country (%)	94.6
- European Union (EU, %)	1.4
- Europe outside EU (%)	1.1
- Outside Europe (%)	2.9
Body Mass Index (kg/m^2^)	26.3 (4.3)
Duration of diabetes (years)	23.4 (15.0)
Smoking (%)	11.8
Disease history	
- CVD (%)	30.0
- Eye disease (%)	22.4
- Lower extremity complication/s (%)	1.4
- Renal failure (%)	0.7
- Atrial fibrillation (%)	1.9
- Depression (%)	2.1
- Other psychiatric condition(s) (%)	2.3
Prescribed insulin pump (%)	15.2
Disability pension or sick-leave last year before inclusion (%)	12.1
HbA1c	64.5 (13.6)
eGFR	82.8 (19.8)
5-year CVD risk (%)	8.5

Sociodemographic factors included sex, age, educational level (stratified in three levels), marital status (four categories) and region of birth (Nordic countries, EU, non-EU Europe, outside Europe). Education level was selected as case-mix factor instead of income level, as previous research point to education being of higher explanatory value regarding diabetes care[18]. Prevalence of additional relevant case-mix factors was identified via administrative systems and the NDR.

Identification of comorbidities was based on regional administrative systems via diagnosis (ICD-10, main or secondary) and procedure codes from two years of inpatient and/or outpatient medical records history. Codes for identification can be found in supplementary S1 Table. BMI, duration of diabetes and smoking habits were identified in the NDR.

### Statistical analysis

Multivariate regression analysis was performed for each of the three study variables. The dependent variables–HbA1c level, change in eGFR and development of risk for cardiovascular events–were treated as continuous variables and analyzed using ordinary least squares (OLS) regression. BMI and duration of diabetes were modelled as continuous variables. The study variables’ association with age was modelled with age groups. Modelling using restricted cubic splines in accordance with previous research[10,19] was also tested. The full set of case-mix factors were used in all three regression models. To assess the impact of these factors on the study variables, regression analysis was adjusted for clustering of health outcomes within individual patients when computing the 95% confidence intervals of each case-mix factor’s effect on the outcome. In addition, as a sensitivity analysis, mixed-effects models were applied with individual identity number as grouping variable, to control for non-independence among each individual’s repeated observations. These results are presented in the supplementary material.

## Results

The study population consisted of 16,367 one-year episodes (11,947 unique prevalent T1D patients) during 2010–2011 with available information in administrative systems and the NDR (included 70.3% of all individuals with T1D identified in administrative systems). Within the time limits stated, 16,367 episodes had HbA1c level registered, 9,593 (7,068 patients) had input values for computing change in one-year eGFR registered, and 978 (725 patients) had all input values for computing one-year change in CVD risk score. Modelling with restricted cubic splines instead of age groups did not show higher degree of determination. Descriptive statistics of the study population are presented in Table 1.

### Determinants of absolute HbA1c levels

**Fig 1:**
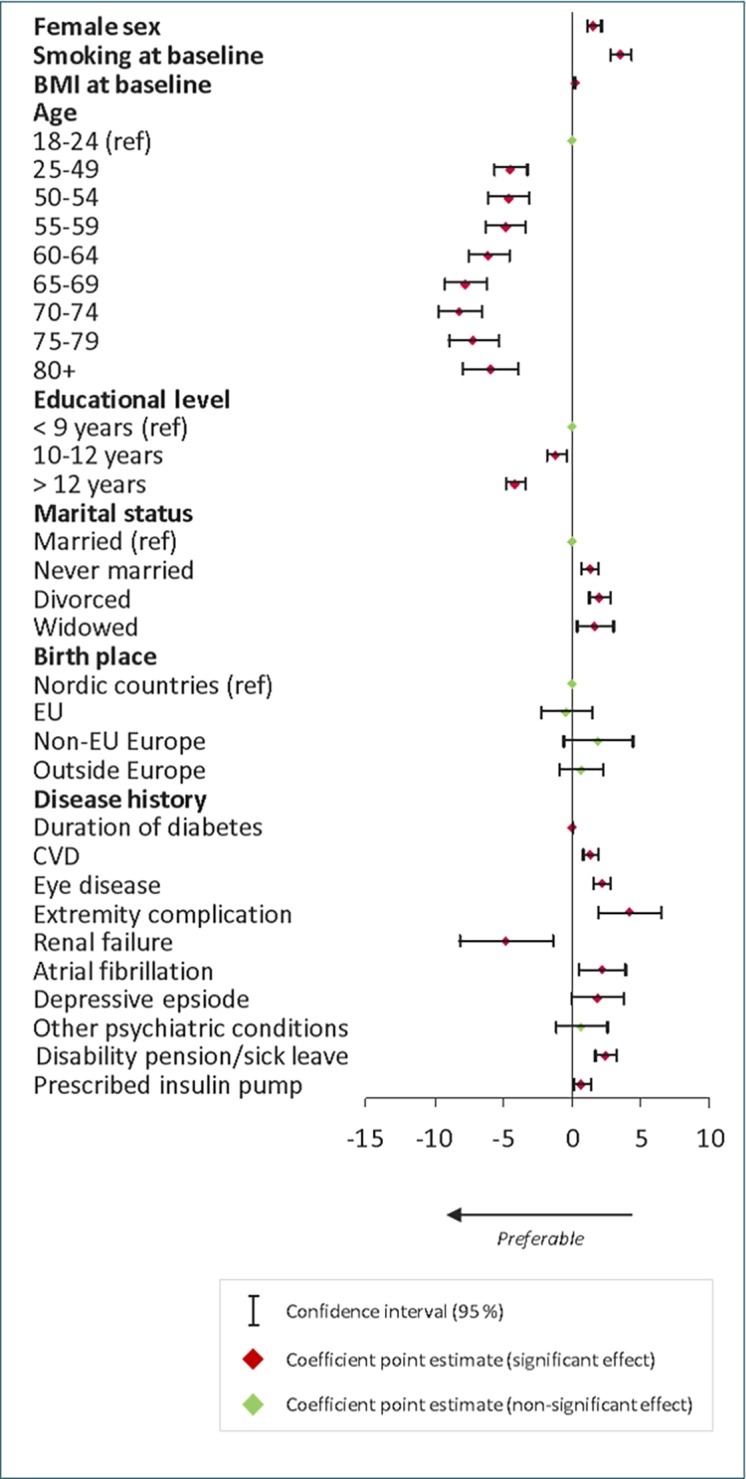
Multivariate regression analysis on HbA1c levels. Coefficient point estimates and their 95% confidence intervals.

Results from the multivariate regression analysis on HbA1c level are presented in Fig 1. Women had higher HbA1c levels than men (p<0.001). Glucose control varied between age groups; patients older than 24 years showed significantly better control than younger patients (p<0.001). Patients aged 70–74 showed best glucose control. Higher education was consistently associated with lower levels of HbA1c; high school education versus comprehensive school (p = 0.002), and college/university degree versus high school/comprehensive school (p<0.001).

Being married was associated with significantly lower HbA1c levels compared to never married (p<0.001), divorced (p<0.001) and widowed (p = 0.009) individuals. No association was found between HbA1c levels and being born outside the Nordic countries, regardless if born in other EU countries or outside of Europe. A history (previous two years) of diabetes-related complications were associated with higher levels of HbA1c (p<0.001). The exception to this pattern was patients with renal failure, who showed significantly lower HbA1c levels (p = 0.006). Smoking (p<0.001), duration of diabetes (per year, p = 0.001) and BMI (per unit, p<0.001) were all individually associated with significantly higher levels of HbA1c. Interestingly, insulin pump prescription was associated with higher HbA1c levels (p = 0.024) as was history of disability pension and/or sick-leave (p<0.001). Coefficients are presented in supplementary tables. Mixed-effects model did not show significant deviations from the original results (S5 Table).

### Determinants of one-year change in estimated glomerular filtration Rate

**Fig 2:**
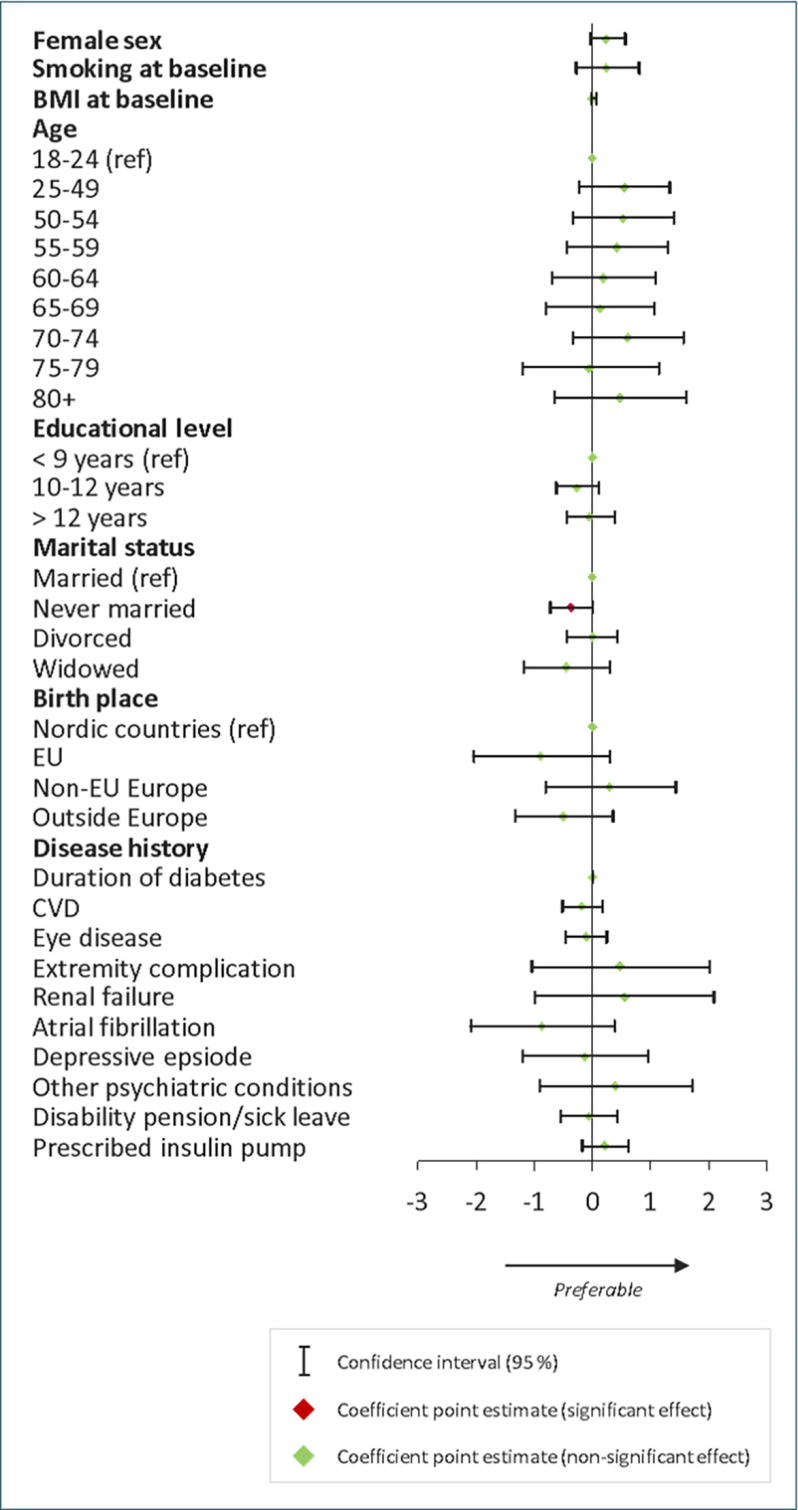
Multivariate regression analysis on one-year change in eGFR. Coefficient point estimates and their 95% confidence intervals.

Average eGFR at baseline was 81.4 ml/min per 1.73 m^2^ and the average one-year change in eGFR was -0.43 ml/min per 1.73 m^2^ per year. Results from the multivariate regression analysis on change in eGFR are presented in Fig 2.

There was no statistically significant association of one-year change in eGFR and educational level. However, never being married was associated with worse development of eGFR compared to being married (p = 0.046). Region of birth was not associated with differences in one-year change of eGFR. Coefficients are presented in supplementary tables. Mixed-effects model did not show significant deviations from the original results (S6 Table).

### Determinants of one-year change in estimated 5-year risk of cardiovascular disease

**Fig 3:**
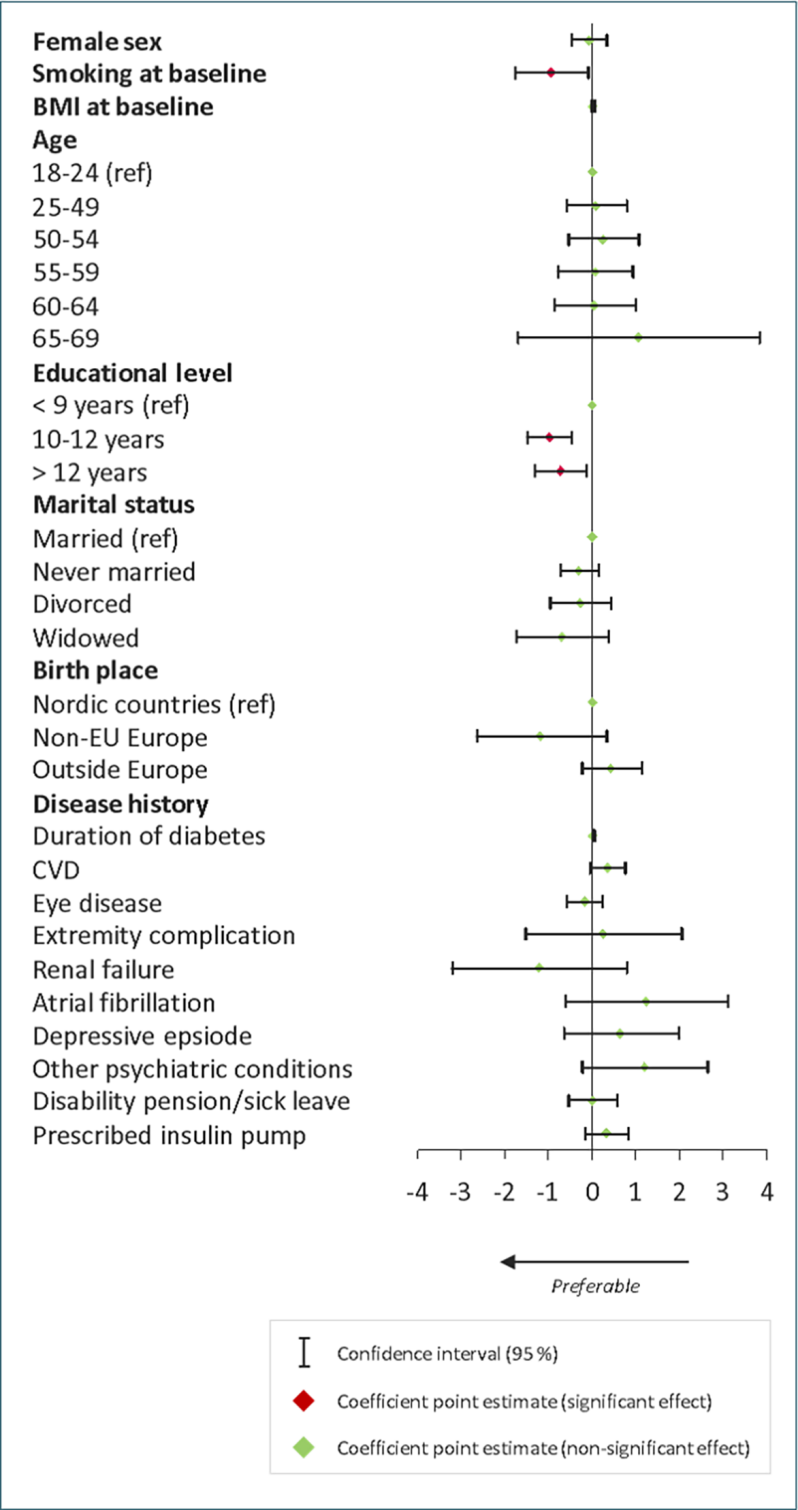
Multivariate regression analysis on one-year change in CVD risk score. Coefficient point estimates and their 95% confidence intervals.

The average baseline CVD risk was 8.6% and the average one-year change was +0.54 percentage points. Results from the multivariate regression analysis on estimated 5-year CVD risk are presented in Fig 3.

Comprehensive school education or lower (≤ 9 years) was associated with larger increase in CVD risk (p<0.001), whilst the association between previous CVD and one-year change of CVD risk was non-significant (p = 0.099). Being a current smoker in the beginning of the follow-up period was associated with lower increase in CVD risk score (p = 0.029) whilst the absolute CVD risk score after one year was significantly higher among smokers (data not shown). Coefficients are presented in supplementary tables. Mixed-effects model did not show significant deviations from the original results (S7 Table).

For analysis of predicted 5-year risk the number of observations was limited due to missing data on individual risk factors such as total/HDL cholesterol ratio and creatinine level (978 observations compared to the original 16,367 observations). To estimate the impact of the significant share of missing data, a comparative analysis was performed, showing no notable differences compared to the original study population (data not shown).

## Discussion

The present study showed that patients’ sociodemographic profile seems to have a strong impact on risk factor control in T1D. Within the study population, HbA1c levels varied significantly with age, sex, educational level and marital status (in addition to previous complications, comorbidities, smoking and BMI level). These systematic differences may stem from different levels of perception and understanding of the T1D diagnosis, its impact on health and measures to take regarding lifestyle and diet, for enhancing short-term and long-term health and avoidance of complications. The results highlight the importance of patient-centred education, acknowledging patients’ differences in social stability and demographic profiles.

Results from this study are in line with previous studies on the subject; consideration to social and demographic determinants of health in individuals with T1D are important. Material and social deprivation is a strong predictor.[20] Hill et al. put forward disengagement from treatment as a potential mediator; with higher levels of education and socioeconomic status come better understanding of treatment.[21] Young adults were at higher risk for higher HbA1c, shown also by Campbell et al.[22] Within this study, country of birth did not stand out as a strong explanatory factor, pointing to that the Swedish healthcare system seemed able to handle such a possible barrier in seeking healthcare. With increasing rates of migration this is an indicator of continuously high importance to follow.

Complications (e.g. extremity, eye, cardiovascular) were associated with higher levels of HbA1c, however, it is unclear whether it stands for lower treatment activity or less successful treatment regimes. Association between renal failure and lower levels may be due to tight monitoring but could partly be due to a decreased insulin degradation with decreasing renal capacity, i.e. the patient remains on an unchanged insulin dose whilst the kidney function decreases, thus potentially indicating an increased risk of hypoglycaemia.[23] Insulin pump prescription was associated with higher levels which may be explained by indication bias (prescription of insulin pump is generally due to bad historic blood glucose control), although no externally valid conclusions can be drawn. Engagement in work or studying have previously been presented as associated with lower HbA1c levels, which is in line with findings from the present study (disability pension/sick-leave was associated with higher levels as was lower educational level). In that context, this study contributes with a larger and on average older study sample.[24] An advantage of this study compared to previous studies in general, was the ability to adjust results for a broad range of factors.

Studying the absolute levels of health outcomes in diabetes conveys the historic quality of care, whereas incremental changes over time measure the quality of care for a specific period. The course of diabetes over one-year cycles may be viewed as short, but it is still of importance as each year of care in T1D incrementally impacts the patient’s health. For the one-year T1D care cycle, a few differences were identified in terms of change in health outcome indicators (eGFR and CVD risk score); being married was associated with lower pace of kidney function deterioration (one-year eGFR change) compared to never married, and educational level was associated with differences in one-year change of CVD risk score. Hence, discrepancies in CVD risk associated to differences in e.g. social or economic conditions continuously increase over time, which is alarming. Further, the present study found that the estimated one-year CVD risk elevation in T1D patients was larger for patients with lower educational level than for patients with previous CVD (the latter was not significantly associated with higher increase in CVD risk score during the studied period). This is consistent with the hypothesis that more well-educated patients may generally be aware of risks and measures to take for secondary prevention but may also be due to that healthcare delivery methods are generally not modified to identify needs and solutions based on patients’ abilities and condition. This finding also points to the value of evaluating change in risk factors rather than only the absolute risk factor levels. Being a current smoker in the beginning of the follow-up period was associated with lower increase in CVD risk score, which may seem contradictory and has also been subject to research.[25] It may be due to that a significant share of diabetes patients stopped smoking during treatment, decreasing their CVD risk compared to baseline and hence implying a relatively larger decrease compared to others. Another reason could be health personnel’s possibly higher active risk factor management towards smokers.

Differences in one-year changes of health outcome indicators between sociodemographic groups were fewer and smaller compared to the analysis of absolute HbA1c levels between groups, consistent with the fact that diverging effects between sociodemographic groups are incremental and developed over longer periods of time (the study population’s average diabetes duration being 23.4 years). The same pattern could probably be expected for one-year development of HbA1c.

Altogether, the findings indicate that ensuring health equity for individuals with T1D long term requires higher consideration to differences in diabetes management with regards to sociodemographic characteristics, through e.g. patient-centered education and customization of prescriptions and instructions.

### Strengths and limitations

This study was based on observational data available from registries with very high coverage rate [11] and included the majority of Sweden’s prevalent T1D cases at the time. Retrospective registry studies do not allow for control of systematic bias in registering, but in order to control for relevant factors to the extent possible, multivariate regression analyses were adjusted for all available patient-related factors deemed relevant for care and expected outcomes. Furthermore, only individuals with confirmed T1D diagnosis were included. With its combination of several national registers and linkage of data on patient level, the present study enabled case-mix adjustments for a broad range of factors.

For the analysis of CVD risk score the share of missing data was significant (978 observations compared to the original 16,367 observations). It was however still of interest to analyze as a comparative analysis performed showed no notable differences compared to the original study population. Nevertheless, the possibilities to draw externally valid conclusions are smaller. It would have been of interest to prolong the time horizon of changes in health outcomes studied, e.g. a 5-year change in eGFR in addition to the one-year change used in this study. The reason for not prolonging the horizon was data scarcity. Thus, longer-term change in risk factor levels between sociodemographic groups is highly recommended as a topic for future research.

## Conclusions

There is a sociodemographic gradient regarding long-term treatment of T1D; patients with lower educational level or not living with a partner (divorced, widowed or never married) showed significantly higher levels of HbA1c as did patients of female sex and those below 25 years of age. A sociodemographic gradient was detectable to some extent also over shorter increments of time, shown for one-year change of eGFR and 5-year CVD risk respectively.

Based on findings from this study, there is a strong need for higher focus on diabetes management education and secondary prevention directed towards individuals, fit to their sociodemographic characteristics. Furthermore, it could be particularly relevant to monitor risk factors more closely in patients living alone. The findings from this study also stress that in addition to comorbidities and demographic factors (age and sex), educational level and marital status are important factors to take into consideration when evaluating and/or comparing health outcomes between groups of patients or e.g. organizational units.

## Supporting information

S1 Table Codes for identification of diagnoses (ICD-10) and procedures. (DOCX)

S2 Table OLS regression of HbA1c in type 1 diabetes patients (16,367 episodes). Beta coefficients, p-values and 95% confidence intervals.
(DOCX)

S3 Table OLS regression of change in eGFR in type 1 diabetes patients during one year (9,522 episodes). Beta coefficients, p-values and 95% confidence intervals.
(DOCX)

S4 Table OLS regression of change in CVD risk in type 1 diabetes patients during one year (954 episodes). Beta coefficients, p-values and 95% confidence intervals.
(DOCX)

S5 Table Mixed-effects regression of HbA1c in type 1 diabetes patients (16,367 episodes). Beta coefficients, p-values and 95% confidence intervals.
(DOCX)

S6 Table Mixed-effects regression of change in eGFR in type 1 diabetes patients during one year (9,522 episodes). Beta coefficients, p-values and 95% confidence intervals.
(DOCX)

S7 Table Mixed-effects regression of change in CVD risk in type 1 diabetes patients during one year (954 episodes). Beta coefficients, p-values and 95% confidence intervals.
(DOCX)

S1 Fig Flowchart depicting the study population. (TIF)
