# Supplementary material for: Sociodemographic determinants and health outcome variation in individuals with type 1 diabetes mellitus: A register-based study
Source: PLoS One. 2018 Jun 29;13(6):e0199170. doi: 10.1371/journal.pone.0199170 (PMC6025867; doi:10.1371/journal.pone.0199170)
Supplement: S1 Table — (DOCX) [file pone.0199170.s001.docx]

**S1 Table.** Codes for identification of diagnoses (ICD-10) and procedures.

|  | **Codes for identification** | |
| --- | --- | --- |
|  | **Diagnosis (ICD-10)** | **Procedure** |
| Cardiovascular disease | I20-I25, | FNA-FNG |
|  | I61, I63-I64 |  |
| Atrial fibrillation | I48 |  |
| Eye disease | H431 |  |
| Lower extremity complication | E106D, E116D | NHQ, |
|  |  | NGQ09, |
|  |  | NGQ19, |
|  |  | NGQ99 |
| Renal failure | N185, | DR014-DR016, |
|  | Y841, Y992, | DR023-DR024, |
|  | Z491-Z492 | QF006 |
| Depression episode | F32 |  |
| Other psychiatric condition | F0, F1, F2, F7 |  |
|  |  |  |
|  |  |  |
|  |  |  |
|  |  |  |
|  |  |  |
|  |  |  |
