# Supplementary material for: Sociodemographic determinants and health outcome variation in individuals with type 1 diabetes mellitus: A register-based study
Source: PLoS One. 2018 Jun 29;13(6):e0199170. doi: 10.1371/journal.pone.0199170 (PMC6025867; doi:10.1371/journal.pone.0199170)
Supplement: S2 Table — Beta coefficients, p-values and 95% confidence intervals. (DOCX) [file pone.0199170.s002.docx]

**S2 Table.** Multivariate regression of HbA1c in type 1 diabetes patients (16,367 episodes). Beta coefficients, p-values and 95% confidence intervals.

|  |  |  | **95% confidence interval** | |
| --- | --- | --- | --- | --- |
|  | **b** | **P-value** | **Lower limit** | **Upper limit** |
| Female sex | 1.60 | 0.000 | 1.09 | 2.11 |
| Smoker at baseline | 3.53 | 0.000 | 2.72 | 4.34 |
| BMI at baseline | .22 | 0.000 | .16 | .28 |
| Age 18-24 (ref) |  |  |  |  |
| Age 25-49 | -4.47 | 0.000 | -5.72 | -3.22 |
| Age 50-54 | -4.62 | 0.000 | -6.06 | -3.18 |
| Age 55-59 | -4.83 | 0.000 | -6.29 | -3.36 |
| Age 60-64 | -6.10 | 0.000 | -7.59 | -4.61 |
| Age 65-69 | -7.75 | 0.000 | -9.25 | -6.24 |
| Age 70-74 | -8.17 | 0.000 | -9.77 | -6.56 |
| Age 75-79 | -7.17 | 0.000 | -8.97 | -5.37 |
| Age > 80 | -5.95 | 0.000 | -7.96 | -3.94 |
| < 9 years of education (ref) |  |  |  |  |
| 10-12 years of education | -1.16 | 0.001 | -1.85 | -.47 |
| > 12 years of education | -4.11 | 0.000 | -4.85 | -3.36 |
| Married (ref) |  |  |  |  |
| Never married | 1.33 | 0.000 | .72 | 1.94 |
| Divorced | 1.96 | 0.000 | 1.19 | 2.73 |
| Widowed | 1.70 | 0.011 | .38 | 3.01 |
| Born within the Nordic countries (ref) |  |  |  |  |
| Born within the EU | -.46 | 0.634 | -2.33 | 1.42 |
| Born within Europe, not EU | 1.94 | 0.132 | -.58 | 4.46 |
| Born outside Europe | .64 | 0.439 | -.98 | 2.25 |
| Duration of diabetes | .03 | 0.000 | .02 | .05 |
| Previous CVD | 1.36 | 0.000 | .80 | 1.91 |
| Previous eye disease | 2.16 | 0.000 | 1.56 | 2.76 |
| Previous lower extremity compl. | 4.20 | 0.000 | 1.85 | 6.54 |
| Previous renal failure | -4.81 | 0.006 | -8.21 | -1.40 |
| Previous atrial fibrillation | 2.19 | 0.011 | .51 | 3.86 |
| Previous depressive episode | 1.85 | 0.059 | -.07 | 3.77 |
| Previous other psychiatric conditions | .68 | 0.477 | -1.19 | 2.56 |
| Disability pension/sick leave | 2.42 | 0.000 | 1.65 | 3.19 |
| Prescribed insulin pump | .72 | 0.025 | .09 | 1.35 |
| Constant | 61.44 | 0.000 | 59.26 | 63.62 |
