# Supplementary material for: Sociodemographic determinants and health outcome variation in individuals with type 1 diabetes mellitus: A register-based study
Source: PLoS One. 2018 Jun 29;13(6):e0199170. doi: 10.1371/journal.pone.0199170 (PMC6025867; doi:10.1371/journal.pone.0199170)
Supplement: S3 Table — Beta coefficients, p-values and 95% confidence intervals. (DOCX) [file pone.0199170.s003.docx]

**S3 Table.** Multivariate regression of change in eGFR in type 1 diabetes patients during one year (9,522 episodes). Beta coefficients, p-values and 95% confidence intervals.

|  |  |  | **95% confidence interval** | |
| --- | --- | --- | --- | --- |
|  | **b** | **P-value** | **Lower limit** | **Upper limit** |
| Female sex | 0.24 | 0.126 | -0.07 | 0.56 |
| Smoker | 0.26 | 0.338 | -0.28 | 0.80 |
| BMI | 0.01 | 0.782 | -0.03 | 0.04 |
| Age 18-24 (ref) |  |  |  |  |
| Age 25-49 | 0.58 | 0.143 | -0.20 | 1.35 |
| Age 50-54 | 0.55 | 0.215 | -0.32 | 1.42 |
| Age 55-59 | 0.43 | 0.333 | -0.44 | 1.31 |
| Age 60-64 | 0.19 | 0.674 | -0.70 | 1.09 |
| Age 65-69 | 0.14 | 0.775 | -0.81 | 1.09 |
| Age 70-74 | 0.63 | 0.186 | -0.30 | 1.57 |
| Age 75-79 | 0.04 | 0.944 | -1.12 | 1.21 |
| Age > 80 | 0.63 | 0.266 | -0.48 | 1.75 |
| < 9 years of education (ref) |  |  |  |  |
| 10-12 years of education | -0.24 | 0.209 | -0.62 | 0.13 |
| > 12 years of education | -0.03 | 0.900 | -0.44 | 0.39 |
| Married (ref) |  |  |  |  |
| Never married | -0.37 | 0.047 | -0.74 | -0.01 |
| Divorced | -0.02 | 0.911 | -0.46 | 0.41 |
| Widowed | -0.54 | 0.144 | -1.27 | 0.19 |
| Born within the Nordic countries (ref) | |  |  |  |
| Born within the EU | -0.69 | 0.251 | -1.86 | 0.49 |
| Born within Europe, not EU | 0.32 | 0.571 | -0.80 | 1.45 |
| Born outside Europe | -0.56 | 0.192 | -1.40 | 0.28 |
| Duration of diabetes | 0.00 | 0.835 | -0.01 | 0.01 |
| Previous CVD | -0.17 | 0.319 | -0.51 | 0.17 |
| Previous eye disease | -0.11 | 0.524 | -0.46 | 0.24 |
| Previous lower extremity compl. | 0.58 | 0.456 | -0.94 | 2.10 |
| Previous renal failure | 0.48 | 0.547 | -1.07 | 2.02 |
| Previous atrial fibrillation | -0.96 | 0.130 | -2.21 | 0.28 |
| Previous depressive episode | -0.12 | 0.829 | -1.21 | 0.97 |
| Previous other psychiatric conditions | 0.38 | 0.576 | -0.95 | 1.70 |
| Disability pension/sick leave | -0.06 | 0.817 | -0.54 | 0.43 |
| Prescribed insulin pump | 0.21 | 0.311 | -0.19 | 0.61 |
| Constant | -0.74 | 0.259 | -2.02 | 0.55 |
