# Supplementary material for: Sociodemographic determinants and health outcome variation in individuals with type 1 diabetes mellitus: A register-based study
Source: PLoS One. 2018 Jun 29;13(6):e0199170. doi: 10.1371/journal.pone.0199170 (PMC6025867; doi:10.1371/journal.pone.0199170)
Supplement: S4 Table — Beta coefficients, p-values and 95% confidence intervals. (DOCX) [file pone.0199170.s004.docx]

**S4 Table.** Multivariate regression of change in CVD risk in type 1 diabetes patients during one year (954 episodes). Beta coefficients, p-values and 95% confidence intervals.

|  |  |  | **95% confidence interval** | |
| --- | --- | --- | --- | --- |
|  | **B** | **P-value** | **Lower limit** | **Upper limit** |
| Female sex | -0.03 | 0.904 | -0.44 | 0.39 |
| Smoker at baseline | -0.91 | 0.043 | -1.80 | -0.03 |
| BMI at baseline | -0.01 | 0.768 | -0.05 | 0.04 |
| Age 18-24 (ref) |  |  |  |  |
| Age 25-49 | 0.10 | 0.775 | -0.56 | 0.75 |
| Age 50-54 | 0.33 | 0.422 | -0.47 | 1.13 |
| Age 55-59 | 0.11 | 0.803 | -0.75 | 0.97 |
| Age 60-64 | 0.06 | 0.903 | -0.86 | 0.97 |
| Age 65-69 | 1.14 | 0.419 | -1.62 | 3.89 |
| Age 70-74 |  |  |  |  |
| Age 75-79 |  |  |  |  |
| Age > 80 |  |  |  |  |
| < 9 years of education (ref) |  |  |  |  |
| 10-12 years of education | -1.01 | 0.000 | -1.55 | -0.47 |
| > 12 years of education | -0.74 | 0.016 | -1.35 | -0.14 |
| Married (ref) |  |  |  |  |
| Never married | -0.26 | 0.248 | -0.70 | 0.18 |
| Divorced | -0.32 | 0.371 | -1.03 | 0.38 |
| Widowed | -0.75 | 0.170 | -1.81 | 0.32 |
| Born within the Nordic countries (ref) |  |  |  |  |
| Born within the EU |  |  |  |  |
| Born within Europe, not EU | -1.27 | 0.117 | -2.86 | 0.32 |
| Born outside Europe | 0.46 | 0.194 | -0.24 | 1.17 |
| Duration of diabetes | 0.01 | 0.140 | 0.00 | 0.03 |
| Previous CVD | 0.31 | 0.147 | -0.11 | 0.72 |
| Previous eye disease | -0.14 | 0.521 | -0.57 | 0.29 |
| Previous lower extremity compl. | 0.30 | 0.750 | -1.55 | 2.14 |
| Previous renal failure | -1.18 | 0.249 | -3.20 | 0.83 |
| Previous atrial fibrillation | 1.30 | 0.171 | -0.56 | 3.17 |
| Previous depressive episode | 0.91 | 0.187 | -0.44 | 2.25 |
| Previous other psychiatric conditions | 0.62 | 0.287 | -0.53 | 1.77 |
| Disability pension/sick leave | -0.01 | 0.963 | -0.59 | 0.57 |
| Prescribed insulin pump | 0.34 | 0.174 | -0.15 | 0.84 |
| Constant | 0.86 | 0.226 | -0.53 | 2.25 |
