# Supplementary material for: Sociodemographic determinants and health outcome variation in individuals with type 1 diabetes mellitus: A register-based study
Source: PLoS One. 2018 Jun 29;13(6):e0199170. doi: 10.1371/journal.pone.0199170 (PMC6025867; doi:10.1371/journal.pone.0199170)
Supplement: S5 Table — Beta coefficients, p-values and 95% confidence intervals. (DOCX) [file pone.0199170.s005.docx]

**S5 Table.** Mixed-effects regression of HbA1c in type 1 diabetes patients (16,367 episodes). Beta coefficients, p-values and 95% confidence intervals.

|  |  |  | **95% confidence interval** | |
| --- | --- | --- | --- | --- |
|  | **b** | **P-value** | **Lower limit** | **Upper limit** |
| Female sex | 1.67 | 0.00 | 1.19 | 2.16 |
| Smoker at baseline | 3.22 | 0.00 | 2.56 | 3.89 |
| BMI at baseline | 0.2 | 0.00 | 0.15 | 0.25 |
| Age 18-24 (ref) |  |  |  |  |
| Age 25-49 | -3.89 | 0.00 | -4.87 | -2.91 |
| Age 50-54 | -3.89 | 0.00 | -5.08 | -2.71 |
| Age 55-59 | -4.29 | 0.00 | -5.51 | -3.08 |
| Age 60-64 | -5.41 | 0.00 | -6.64 | -4.18 |
| Age 65-69 | -6.77 | 0.00 | -8.05 | -5.5 |
| Age 70-74 | -7.45 | 0.00 | -8.85 | -6.06 |
| Age 75-79 | -6.32 | 0.00 | -7.9 | -4.75 |
| Age > 80 | -4.76 | 0.00 | -6.55 | -2.97 |
| < 9 years of education (ref) |  |  |  |  |
| 10-12 years of education | -1.13 | 0.00 | -1.76 | -0.49 |
| > 12 years of education | -4.35 | 0.00 | -5.05 | -3.64 |
| Married (ref) |  |  |  |  |
| Never married | 1.41 | 0.00 | 0.84 | 1.98 |
| Divorced | 1.71 | 0.00 | 1,00 | 2.42 |
| Widowed | 1.25 | 0.06 | -0.04 | 2.54 |
| Born within the Nordic countries (ref) |  |  |  |  |
| Born within the EU | -0.35 | 0.72 | -2.32 | 1.61 |
| Born within Europe, not EU | 2.24 | 0.04 | 0.06 | 4.42 |
| Born outside Europe | 0.75 | 0.29 | -0.63 | 2.14 |
| Duration of diabetes | 0.04 | 0.00 | 0.02 | 0.05 |
| Previous CVD | 0.84 | 0.00 | 0.35 | 1.33 |
| Previous eye disease | 1.97 | 0.00 | 1.39 | 2.56 |
| Previous lower extremity compl. | 3.27 | 0.00 | 1.57 | 4.96 |
| Previous renal failure | -4.26 | 0.00 | -6.6 | -1.93 |
| Previous atrial fibrillation | 1.66 | 0.03 | 0.12 | 3.19 |
| Previous depressive episode | 0.55 | 0.43 | -0.81 | 1.9 |
| Previous other psychiatric conditions | 0.95 | 0.17 | -0.41 | 2.31 |
| Disability pension/sick leave | 2.01 | 0.00 | 1.39 | 2.63 |
| Prescribed insulin pump | 0.71 | 0.03 | 0.08 | 1.33 |
| Constant | 61.72 | 0.00 | 59.89 | 63.55 |
