# Supplementary material for: Sociodemographic determinants and health outcome variation in individuals with type 1 diabetes mellitus: A register-based study
Source: PLoS One. 2018 Jun 29;13(6):e0199170. doi: 10.1371/journal.pone.0199170 (PMC6025867; doi:10.1371/journal.pone.0199170)
Supplement: S6 Table — Beta coefficients, p-values and 95% confidence intervals. (DOCX) [file pone.0199170.s006.docx]

**S6 Table.** Mixed-effects regression of change in eGFR in type 1 diabetes patients during one year (9,522 episodes). Beta coefficients, p-values and 95% confidence intervals.

|  |  |  | **95% confidence interval** | |
| --- | --- | --- | --- | --- |
|  | **b** | **P-value** | **Lower limit** | **Upper limit** |
| Female sex | 0.25 | 0.13 | -0.07 | 0.56 |
| Smoker at baseline | 0.25 | 0.32 | -0.24 | 0.74 |
| BMI at baseline | 0.01 | 0.73 | -0.03 | 0.04 |
| Age 18-24 (ref) |  |  |  |  |
| Age 25-49 | 0.54 | 0.15 | -0.19 | 1.27 |
| Age 50-54 | 0.53 | 0.23 | -0.33 | 1.4 |
| Age 55-59 | 0.42 | 0.35 | -0.46 | 1.31 |
| Age 60-64 | 0.2 | 0.66 | -0.69 | 1.08 |
| Age 65-69 | 0.12 | 0.8 | -0.79 | 1.03 |
| Age 70-74 | 0.61 | 0.23 | -0.38 | 1.59 |
| Age 75-79 | -0.04 | 0.94 | -1.14 | 1.06 |
| Age > 80 | 0.47 | 0.44 | -0.73 | 1.67 |
| < 9 years of education (ref) |  |  |  |  |
| 10-12 years of education | -0.26 | 0.21 | -0.65 | 0.14 |
| > 12 years of education | -0.04 | 0.84 | -0.49 | 0.4 |
| Married (ref) |  |  |  |  |
| Never married | -0.37 | 0.05 | -0.75 | 0.00 |
| Divorced | -0.01 | 0.96 | -0.47 | 0.45 |
| Widowed | -0.46 | 0.26 | -1.25 | 0.34 |
| Born within the Nordic countries (ref) |  |  |  |  |
| Born within the EU | -0.89 | 0.17 | -2.16 | 0.38 |
| Born within Europe, not EU | 0.29 | 0.66 | -1.00 | 1.59 |
| Born outside Europe | -0.5 | 0.28 | -1.39 | 0.4 |
| Duration of diabetes | 0.00 | 0.71 | -0.01 | 0.01 |
| Previous CVD | -0.19 | 0.3 | -0.54 | 0.17 |
| Previous eye disease | -0.11 | 0.56 | -0.48 | 0.26 |
| Previous lower extremity compl. | 0.47 | 0.43 | -0.7 | 1.64 |
| Previous renal failure | 0.54 | 0.55 | -1.22 | 2.31 |
| Previous atrial fibrillation | -0.87 | 0.1 | -1.93 | 0.18 |
| Previous depressive episode | -0.14 | 0.8 | -1.2 | 0.93 |
| Previous other psychiatric conditions | 0.4 | 0.45 | -0.65 | 1.45 |
| Disability pension/sick leave | -0.06 | 0.81 | -0.53 | 0.42 |
| Prescribed insulin pump | 0.21 | 0.35 | -0.22 | 0.63 |
| Constant | -0.68 | 0.28 | -1.91 | 0.56 |
