# Supplementary material for: Sociodemographic determinants and health outcome variation in individuals with type 1 diabetes mellitus: A register-based study
Source: PLoS One. 2018 Jun 29;13(6):e0199170. doi: 10.1371/journal.pone.0199170 (PMC6025867; doi:10.1371/journal.pone.0199170)
Supplement: S7 Table — Beta coefficients, p-values and 95% confidence intervals. (DOCX) [file pone.0199170.s007.docx]

**S7 Table.** Mixed-effects regression of change in CVD risk in type 1 diabetes patients during one year (954 episodes). Beta coefficients, p-values and 95% confidence intervals.

|  |  |  | **95% confidence interval** | |
| --- | --- | --- | --- | --- |
|  | **b** | **P-value** | **Lower limit** | **Upper limit** |
| Female sex | -0.09 | 0.72 | -0.55 | 0.38 |
| Smoker at baseline | -0.94 | 0.01 | -1.61 | -0.27 |
| BMI at baseline | -0.01 | 0.70 | -0.06 | 0.04 |
| Age 18-24 (ref) |  |  |  |  |
| Age 25-49 | 0.07 | 0.96 | -2.50 | 2.65 |
| Age 50-54 | 0.24 | 0.86 | -2.40 | 2.89 |
| Age 55-59 | 0.06 | 0.96 | -2.58 | 2.71 |
| Age 60-64 | 0.05 | 0.97 | -2.61 | 2.71 |
| Age 65-69 | 1.06 | 0.49 | -1.97 | 4.09 |
| Age 70-74 |  |  |  |  |
| Age 75-79 |  |  |  |  |
| Age > 80 |  |  |  |  |
| < 9 years of education (ref) |  |  |  |  |
| 10-12 years of education | -0.99 | 0.00 | -1.54 | -0.45 |
| > 12 years of education | -0.75 | 0.03 | -1.40 | -0.09 |
| Married (ref) |  |  |  |  |
| Never married | -0.30 | 0.25 | -0.81 | 0.21 |
| Divorced | -0.29 | 0.37 | -0.92 | 0.35 |
| Widowed | -0.70 | 0.51 | -2.78 | 1.37 |
| Born within the Nordic countries (ref) |  |  |  |  |
| Born within the EU |  |  |  |  |
| Born within Europe, not EU | -1.18 | 0.21 | -3.01 | 0.66 |
| Born outside Europe | 0.44 | 0.42 | -0.61 | 1.49 |
| Duration of diabetes | 0.02 | 0.08 | 0.00 | 0.04 |
| Previous CVD | 0.34 | 0.16 | -0.13 | 0.80 |
| Previous eye disease | -0.19 | 0.41 | -0.65 | 0.27 |
| Previous lower extremity compl. | 0.26 | 0.65 | -0.83 | 1.34 |
| Previous renal failure | -1.22 | 0.06 | -2.51 | 0.06 |
| Previous atrial fibrillation | 1.23 | 0.14 | -0.39 | 2.85 |
| Previous depressive episode | 0.65 | 0.41 | -0.9 | 2.19 |
| Previous other psychiatric conditions | 1.19 | 0.14 | -0.4 | 2.78 |
| Disability pension/sick leave | 0.00 | 1.00 | -0.53 | 0.53 |
| Prescribed insulin pump | 0.31 | 0.28 | -0.25 | 0.86 |
| Constant | 0.96 | 0.53 | -2.03 | 3.94 |
